# Standardized, systemic phenotypic analysis of *Slc12a1*^*I299F*^ mutant mice

**DOI:** 10.1186/s12929-014-0068-0

**Published:** 2014-08-02

**Authors:** Elisabeth Kemter, Birgit Rathkolb, Lore Becker, Ines Bolle, Dirk H Busch, Claudia Dalke, Ralf Elvert, Jack Favor, Jochen Graw, Wolfgang Hans, Boris Ivandic, Svetoslav Kalaydjiev, Thomas Klopstock, Ildikó Rácz, Jan Rozman, Anja Schrewe, Holger Schulz, Andreas Zimmer, Helmut Fuchs, Valérie Gailus-Durner, Martin Hrabé de Angelis, Eckhard Wolf, Bernhard Aigner

**Affiliations:** 1 Chair for Molecular Animal Breeding and Biotechnology, and Laboratory for Functional Genome Analysis (LAFUGA), Gene Center, LMU Munich, Munich, 81377, Germany; 2 German Mouse Clinic, Institute of Experimental Genetics, Helmholtz Zentrum München, German Research Center for Environmental Health, Neuherberg, 85764, Germany; 3 Institute of Medical Microbiology, Immunology, and Hygiene, TU Munich, Munich, 81675, Germany; 4 Institute of Developmental Genetics, Helmholtz Zentrum München, German Research Center for Environmental Health, Neuherberg, 85764, Germany; 5 Institute of Human Genetics, Helmholtz Zentrum München, German Research Center for Environmental Health, Neuherberg, 85764, Germany; 6 Department of Medicine III, Division of Cardiology, University of Heidelberg, Heidelberg, 69120, Germany; 7 Department of Neurology, Friedrich-Baur-Institut, LMU Munich, Munich, 80336, Germany; 8 German Center for Vertigo and Balance Disorders, University Hospital Munich, Campus Grosshadern, Munich, 81377, Germany; 9 Laboratory of Molecular Neurobiology, Department of Psychiatry, University of Bonn, Bonn, 53105, Germany; 10 Member of German Center for Diabetes Research (DZD), Neuherberg, 85764, Germany; 11 Institute of Epidemiology I, Helmholtz Zentrum München, German Research Center for Environmental Health, Neuherberg, 85764, Germany; 12 Chair of Experimental Genetics, Center of Life and Food Sciences Weihenstephan, TU Munich, Freising-Weihenstephan, 85350, Germany

**Keywords:** Animal model, NKCC2, Systematic phenotype analysis

## Abstract

**Background:**

Type I Bartter syndrome is a recessive human nephropathy caused by loss-of-function mutations in the *SLC12A1* gene coding for the Na^+^-K^+^-2Cl^−^ cotransporter NKCC2. We recently established the mutant mouse line *Slc12a1*^*I299F*^ exhibiting kidney defects highly similar to the late-onset manifestation of this hereditary human disease. Besides the kidney defects, low blood pressure and osteopenia were revealed in the homozygous mutant mice which were also described in humans. Beside its strong expression in the kidney, NKCC2 has been also shown to be expressed in other tissues in rodents i.e. the gastrointestinal tract, pancreatic beta cells, and specific compartments of the ear, nasal tissue and eye.

**Results:**

To examine if, besides kidney defects, further organ systems and/or metabolic pathways are affected by the *Slc12a1*^*I299F*^ mutation as primary or secondary effects, we describe a standardized, systemic phenotypic analysis of the mutant mouse line *Slc12a1*^*I299F*^ in the German Mouse Clinic. *Slc12a1*^*I299F*^ homozygous mutant mice and *Slc12a1*^*I299F*^ heterozygous mutant littermates as controls were tested at the age of 4–6 months. Beside the already published changes in blood pressure and bone metabolism, a significantly lower body weight and fat content were found as new phenotypes for *Slc12a1*^*I299F*^ homozygous mutant mice. Small additional effects included a mild erythropenic anemia in homozygous mutant males as well as a slight hyperalgesia in homozygous mutant females. For other functions, such as immunology, lung function and neurology, no distinct alterations were observed.

**Conclusions:**

In this systemic analysis no clear primary effects of the *Slc12a1*^*I299F*^ mutation appeared for the organs other than the kidneys where *Slc12a1* expression has been described. On the other hand, long-term effects additional and/or secondary to the kidney lesions might also appear in humans harboring *SLC12A1* mutations.

## Background

The solute carrier family 12 member 1 (*SLC12A1*) gene encodes the bumetanide-sensitive Na^+^-K^+^-2Cl^−^ cotransporter NKCC2. The electroneutral cotransporter NKCC2 belongs to the Na^+^-dependent subgroup of the solute carrier 12 (SLC12) family of transporters. They are membrane-bound proteins and mediate the electroneutral movement of Na^+^ and K^+^, tightly coupled to the movement of Cl^−^ across cell membranes from the outside to the inside of cells. NKCC2 is mainly expressed in the kidney in the apical membrane of the cells of the thick ascending limb of Henle’s loop (TALH) and of the macula densa. It is responsible for one of the major salt transport pathways in the kidney. It is involved in the establishment and maintenance of salt accumulation in the medulla which is required for the urine concentration mechanism and, thus, for the maintenance of body salt and water homeostasis. In consequence, NKCC2 influences salt reabsorption, regulation of acid–base and divalent mineral cation metabolism. Further, by sensing luminal Cl^−^ concentration to macula densa cells, NKCC2 function is essential for renin release and for tubuloglomerular feedback mechanism in the kidney matching glomerular filtration to tubular reabsorption. NKCC2 is the main pharmacological target of loop diuretics like bumetanide or furosemide [1]–[3].

Several studies on the effect of sex steroids on NKCC2 abundance in rodent kidneys yielded variable results ([4] and refs therein). Beside its strong expression in the kidney, SLC12A1 in rodents was also shown to be expressed in other tissues i.e. the gastrointestinal tract [5], pancreatic beta cells [6], and specific compartments of the ear [7], nasal tissue [8] and eye [9], although at lower levels when compared to kidney.

In patients suffering from type I Bartter syndrome (antenatal Bartter’s disease, hyperprostaglandin E syndrome), compound heterozygous or homozygous mutations of the *SLC12A1* gene (OMIM 601678) were identified as cause [10]. To date, more than 70 causative mutations of *SLC12A1* were found in patients showing lead symptoms of antenatal Bartter syndrome, Bartter syndrome, and/or reduced blood pressure. Most mutations are missense/nonsense mutations but splice site mutations as well as small insertions/deletions also appeared (http://www.hgmd.org). The mutations result in decreased or absent activity of SLC12A1. NKCC2 acts as dimer, in which the individual subunits transport Na^+^ independently, and there is a huge reserve capacity of ion transport of NKCC2 in the kidney. In consequence, heterozygous mutants show no obvious renal disease phenotype due to the presence of a functional wild-type allele which is sufficient to compensate for the functional inactive mutant transcript. The hereditary recessive disease type I Bartter syndrome in homozygous mutant or compound heterozygous mutant patients is characterized by prenatal onset, a severe salt-wasting state with low blood pressure, metabolic alkalosis with hypokalemia, hyperreninemia, polyuria, hyperprostaglandinuria, and often hypercalciuria. It is complicated by severe dehydration episodes, recurrent vomiting, failure to thrive and growth retardation. Clinical symptoms of type I Bartter syndrome in humans often start to appear before birth, in the last trimenon of pregnancy, leading to polyhydramnios and premature birth due to intrauterine polyuria [2]. However, also patients exhibiting a mild and/or late-onset manifestation of type I Bartter syndrome as a result of a residual function of the mutant NKCC2 transporter were described. This included two siblings with the onset of clinical symptoms during puberty [11] as well as other cases [12].

Recently, rare heterozygous missense mutations of *SLC12A1* were identified in a screen of members of the Framingham Heart Study to act beneficially against the risk of arterial hypertension and death due to cardiovascular disease. These mutant NKCC2 proteins exhibited a reduced ion transport capacity, and this chronic reduction of NKCC2 function was discussed to result in a lower blood pressure and decreased risk of hypertension in otherwise healthy individuals [13]–[16]. Reversely, enhanced activity of NKCC2 has been linked to hypertension and hypertensive disorders in rodents and humans ([3] and refs. therein).

In mice, SLC12A1 has 1095 amino acids. Different full-length splice variants of *Slc12a1* are expressed. The main isoforms A, B and F are derived by differential splicing of one of three mutually exclusive exon cassettes which encode the second transmembrane domain and parts of the adjacent intracellular loop of the protein [17]. To date, various *Slc12a1* knockout mutant mouse models have been published. Mice with a complete lack of the NKCC2 cotransporter obtained by targeted disruption of the *Slc12a1* gene by deletion of promoter sequences and exons 1–3 maintained on the 129/SvEv inbred genetic background were born normally, but died within the first two weeks postpartum due to severe renal failure and metabolic alterations [18]. Two isoform-specific mouse knockouts for SLC12A1 were generated by introducing premature stop codons into the mutually exclusive exon cassettes in the mixed 129/SvEv and C57BL/6 genetic background. Both mouse lines deficient in either NKCC2 isoform A or B exhibited minor phenotypic kidney alterations [19],[20].

In the phenotype-based Munich ENU mouse mutagenesis project using C3HeB/FeJ (C3H) inbred mice as genetic background, several mutant lines were established showing increased plasma urea levels as a parameter indicative of kidney disease [21]. One line showed the mutation *Slc12a1*^*I299F*^ which affects all full-length isoforms of the gene. Homozygous mutant mice are viable and reproduce normally. The markedly increased plasma urea concentration is not due to a general renal failure as plasma creatinine is only slightly elevated, and the creatinine clearance is higher than in wild-type mice. Homozygous mutants show a severe defect in their ability to concentrate urine, leading to increased urine excretion and water consumption. In addition, prostaglandin E as well as calcium and magnesium excretion are markedly increased, and the fractional excretion of uric acid is reduced. In addition, homozygous mutants exhibit hypotension and osteopenia. Thus, they exhibit a late-onset manifestation of type I Bartter syndrome and may represent a new model for the study of this human disease [22]. Heterozygous mutants of line *Slc12a1*^*I299F*^ showed only slight deviations and are highly similar to the wild-type phenotype [22]. This was also observed in *Slc12a1* heterozygous knockout mice [23].

To examine if further organ systems and/or metabolic pathways are affected as primary or secondary effects, we describe a standardized, systemic phenotypic analysis of the mutant mouse line *Slc12a1*^*I299F*^ in the German Mouse Clinic (http://www.mouseclinic.de) covering many vital organ systems and functions. As nephropathy-causing reduced NKCC2 ion transport capacity occurs if both alleles are mutated, homozygous mutant mice were analyzed and compared to healthy heterozygous mutant controls.

## Methods

The recessive mutant line *Slc12a1*^*I299F*^ was established in the Munich ENU mouse mutagenesis project using C3HeB/FeJ (C3H) inbred mice as genetic background [24]. Maintenance of the line comprised the repeated backcross to C3H wild-type mice leading to the subsequent loss of non-causative ENU mutations that were not linked to the *Slc12a1* mutation. The systemic, comprehensive phenotypic analysis was carried out in the German Mouse Clinic at the Helmholtz Zentrum München by using standardized examination protocols (http://www.mouseclinic.de). The analysis covers over 300 parameters in the areas of allergy, behavior, bone and cartilage, cardiovascular analysis, clinical chemistry, energy metabolism, eye analysis and vision, immunology, lung function, molecular phenotyping, neurology, nociception, pathology, and steroid metabolism [25]–[27].

Homozygous mutant mice and heterozygous mutant littermates as controls were tested at defined time points in the German Mouse Clinic (see Additional file 1: Table S1). The analyses used defined numbers of animals (stated in the text of the respective Results section) out of a cohort of 15–17 animals per sex and genotype. Furthermore, an additional cohort of homozygous mutant mice and wild-type littermates were used for selected analyses like clinical chemistry on further age groups [22].

Mouse husbandry was done under a continuously controlled specific pathogen-free (SPF) hygiene standard according to the FELASA recommendations (http://www.felasa.eu) [28].

If not otherwise stated, statistical analysis of data was carried out by Student’s *t*-test. Data are shown as mean ± standard error of the mean. Significant differences are indicated for *P* < 0.05, 0.01, and 0.001.

### Ethics statement

Mouse husbandry and all tests were carried out under the approval of the responsible animal welfare authority (Regierung von Oberbayern, Germany).

## Results

### Phenotypic analysis in the German Mouse Clinic

The recessive mutant line *Slc12a1*^*I299F*^ was established by the detection of increased plasma urea levels in homozygous mutant mice. Plasma urea levels of heterozygous mutants and C3H wild-type controls were similar, and therefore heterozygous mutants were defined as healthy without nephropathy [21],[22]. As disease-causing reduced NKCC2 ion transport capacity occurs if both alleles are mutated, homozygous mutant mice as well as heterozygous mutant littermates as controls were tested in the German Mouse Clinic (see Additional file 1: Table S1).

Phenotypic analysis of mutant mice in the German Mouse Clinic aims at collecting and delivering with free access comprehensive phenome data of a high number of mutant mouse lines in a standardized manner. The phenotype report of mutant line *Slc12a1*^*I299F*^ (see line “HST009”) is deposited online (http://146.107.35.38/phenomap/jsp/annotation/public/phenomap.jsf).

### Additional clinical chemistry analysis

Blood data of 3-month-old homozygous mutants, heterozygous mutants and wild-type controls of line *Slc12a1*^*I299F*^ (n = 10 per genotype and sex) were previously described. They were confirmed by the analysis of 6-month- and 1-year-old mice [22]. The additional analysis of the clinical-chemical and hematological parameters of homozygous mutants vs. heterozygous mutants as controls (n = 15 per genotype and sex) at the age of 17 weeks revealed comparable results. Thus, homozygous mutants of both sexes showed increased values for calcium, creatinine, urea (127 ± 3 mg/dl vs. 61 ± 2 mg/dl in males, 92 ± 4 mg/dl vs. 54 ± 2 mg/dl in females; P < 0.001), alkaline phosphatase (93 ± 4 U/l vs. 84 ± 2 U/l in males, P < 0.05; 120 ± 4 U/l vs. 109 ± 4 U/l in females, not significant; similar but not significant differences were observed in 8–10 mice per genotype and sex as a subgroup of the previously tested mice at the age of 22 weeks), alpha amylase and lipase, as well as decreased values for chloride, whereas the levels for sodium, potassium, inorganic phosphate, total protein, uric acid, cholesterol, triglycerides, glucose, lactate, creatine kinase, alanine aminotransferase (ALT) and aspartate aminotransferase (AST) were not significantly altered. In the hematological analysis at both ages (17 weeks and 22 weeks), male homozygous mutants showed increased values for the white blood cell count, as well as decreased values for the red blood cell count, platelet count, hemoglobin and hematocrit, whereas female homozygous mutants showed no significant differences (Table 1). Thus, a slight erythropenia was revealed in the male homozygous mutants.

**Table 1:** **Hematological analysis of line** ***Slc12a1***^***I299F***^

	**Males**		**Females**	
**Parameter**	**Homozygous mutants**	**Heterozygous mutants**	**Homozygous mutants**	**Heterozygous mutants**
WBC (10^3^/μl)	8.4 ± 0.9^a^	6.4 ± 0.3	7.0 ± 0.2	6.8 ± 0.5
RBC (10^6^/μl)	8.8 ± 0.1^a^	9.1 ± 0.1	9.0 ± 0.2	9.0 ± 0.1
PLT (10^3^/μl)	680 ± 14^c^	760 ± 12	671 ± 21	704 ± 22
HGB (g/dl)	14.2 ± 0.2^b^	14.9 ± 0.1	14.8 ± 0.3	14.5 ± 0.2
HCT (%)	43.5 ± 0.5^b^	46.1 ± 0.4	45.3 ± 0.8	45.0 ± 0.4
MCV (fl)	49.5 ± 0.2^b^	50.4 ± 0.2	50.4 ± 0.3	50.1 ± 0.2
MCH (pg)	16.1 ± 0.1	16.3 ± 0.1	16.4 ± 0.1	16.1 ± 0.1
MCHC (g/dl)	32.6 ± 0.2	32.3 ± 0.1	32.7 ± 0.2	32.3 ± 0.1

WBC, white blood cell count; RBC, red blood cell count; PLT, platelet count; HGB, hemoglobin; HCT, hematocrit; MCV, mean corpuscular volume; MCH, mean corpuscular hemoglobin; MCHC, mean corpuscular hemoglobin concentration.

22-week-old mice were tested. No. per genotype and sex: n = 8–10. Data are presented as mean ± standard error of mean. Student’s *t*-test vs. heterozygous mutants as controls: ^a^*P* < 0.05, ^b^*P* < 0.01, ^c^*P* < 0.001.

### Additional analysis of cardiovascular function

Analysis of cardiovascular function of 3-month-old mice of line *Slc12a1*^*I299F*^ was previously described. Compared to wild-type controls, mean arterial blood pressure as well as systolic and diastolic pressures were significantly decreased in homozygous mutants. Heart rate did not differ significantly between genotypes. In addition, plasma concentration of the N-terminal fragment of the pro-atrial natriuretic peptide (ANP) was significantly increased in homozygous mutants of both genders. ANP is a cardiac hormone predominantly secreted by atrial myocytes in response to cardiac filling pressures. Plasma renin concentration in homozygous mutant mice was significantly lower than in wild-type littermate controls [22].

Additional analysis of 19-week-old homozygous mutants vs. heterozygous mutants as controls (n = 9–11 mice per genotype and sex) for mean arterial blood pressure as well as systolic pressure and diastolic pressure showed the same tendency without reaching statistical significance (Table 2). Compared to a cohort of C3H mice taken from a different breeding colony and maintained under different housing conditions (systolic pressure: 127 ± 2 mm Hg in males and 124 ± 2 mm Hg in females; diastolic pressure: 112 ± 2 mm Hg in males and 109 ± 3 mm Hg in females), the mutants showed low blood pressure values.

**Table 2:** **Cardiovascular analysis of line** ***Slc12a1***^***I299F***^

	**Males**		**Females**	
**Parameter**	**Homozygous mutants**	**Heterozygous mutants**	**Homozygous mutants**	**Heterozygous mutants**
Systolic pressure (mm Hg)	103 ± 3	107 ± 2	105 ± 3	111 ± 3
Diastolic pressure (mm Hg)	86 ± 3	89 ± 2	90 ± 2	93 ± 3
Mean arterial pressure (mm Hg)	91 ± 3	94 ± 2	95 ± 2	99 ± 3
Pulse (bpm)	561 ± 15^a^	602 ± 7	615 ± 11	614 ± 20

19-week-old mice were tested. No. per genotype and sex: n = 9–11. Data are presented as mean ± standard error of mean. Student’s *t*-test vs. heterozygous mutants as controls: ^a^*P* < 0.05.

In addition, electrocardiography was done in the identical 20-week-old mice to analyze quantitative (PQ interval, P-wave duration, QRS-complex duration, QT interval, RR interval, heart rate, JT interval, ST interval, Q amplitude, R amplitude, S amplitude, QRS amplitude) as well as qualitative parameters (arrhythmias). No significant genotype-specific phenotype was revealed between homozygous mutant mice and heterozygous mutant littermates as controls (not shown).

### Additional analysis of dysmorphology

In addition to the previously published differences in bone metabolism in 9 month-old homozygous mutants [22], systemic morphological investigation via visual inspection and X-ray analysis according to standardized protocols were carried out. Fifteen mice of each genotype and sex were analyzed by morphological inspection at the age of 14 weeks. The systematic morphological investigation via visual inspection (growth/weight/body size, eye, coat, skin, vibrissae, extremities including limbs, digits and tail, teeth, ears, musculature, behavior/movement, feeding/drinking, respiratory system, other conspicuous phenotypes) found genotype-specific differences for two parameters. Compared to the heterozygous mutant controls, 10 of the 15 female homozygous mutants analyzed showed a pale colour of the maxillary incisors, which might reflect a mineralization defect via deposition of calcium salts. This was not observed in male homozygous mutants. In addition, two of the 15 male and all 15 female homozygous mutants showed a pale colour of the urine. All other parameters of the visual inspection were inconspicuous in homozygous mutant mice compared to heterozygous mutant littermates as controls.

Hearing ability was examined by the clickbox test using a sound of 20 kHz. In males, all 15 homozygous mutants and all 15 heterozygous mutants showed a normal reaction. In females, 14 homozygous mutants and 14 heterozygous mutants showed a normal reaction, whereas 1 mouse each of the groups of homozygous mutants and heterozygous mutants showed a retarded reaction. Thus, no genotype-specific phenotype was found between homozygous mutants and heterozygous mutants as controls.

Ten males each of homozygous mutants and heterozygous mutants as well as 11 female homozygous mutants and 9 female heterozygous mutants entered the bone density and X-ray analysis at the age of 21 weeks. X-ray analysis of the skeleton (skull shape, mandibles, maxilla, teeth, orbit, number of vertebrae, vertebrae shape, number of ribs, rib shape, scapula, clavicle, pelvis, femur shape, tibia, fibula, humerus, ulna, radius, number of digits, completeness of digits, joints) found no genotype-specific differences between homozygous mutants and heterozygous mutants.

In the dual energy X-ray absorption (DXA) analysis of bone- and weight-related parameters at the age of 21 weeks, bone mineral density (BMD) and bone mineral content (BMC) were decreased in homozygous mutants, however BMD related to the body weight (sBMD) was not significantly changed. Body weight and fat content were significantly decreased in homozygous mutants, and lean content was increased. Differences between homozygous mutants and heterozygous mutant controls in bone parameters might be due to changes in body composition and thus be secondary effects (Table 3). Additional analyses of 9-month-old mice have been already published and showed the significant reduction of the femoral cortical bone mineral density in homozygous mutants compared with wild-type mice, indicating osteopenia [22].

**Table 3:** **Dual energy X-ray absorption (DXA) analysis of bone- and weight-related parameters in line** ***Slc12a1***^***I299F***^**at the age of 21 weeks**

	**Males**		**Females**	
**Parameter**	**Homozygous mutants**	**Heterozygous mutants**	**Homozygous mutants**	**Heterozygous mutants**
Body length (cm)	10.3 ± 0.1	10.3 ± 0.1	10.3 ± 0.1	10.4 ± 0.1
Body weight (g)	33.3 ± 0.5^c^	40.0 ± 1.0	32.1 ± 1.1^a^	35.9 ± 1.4
Lean mass (units)	23.0 ± 1.0^b^	17.1 ± 1.3	18.7 ± 1.0^b^	14.1 ± 0.9
Lean content (units × 100/g)	69.0 ± 2.7^c^	42.7 ± 3.2	59.4 ± 4.4^b^	40.1 ± 3.7
Fat mass (units)	6.7 ± 0.9^c^	18.6 ± 1.4	9.7 ± 1.8^b^	17.7 ± 1.9
Fat content (units × 100/g)	20.2 ± 2.6^c^	46.4 ± 3.1	29.0 ± 4.4^b^	48.2 ± 3.6
BMD (mg/cm^2^)	67 ± 1^c^	75 ± 1	70 ± 1^b^	75 ± 1
sBMD (10^−3^ × cm^−2^)	2.0 ± 0.1	1.9 ± 0.1	2.2 ± 0.04	2.1 ± 0.1
BMC (mg)	890 ± 45^c^	1306 ± 67	966 ± 68^a^	1234 ± 67
Bone content (%)	2.7 ± 0.1^a^	3.3 ± 0.2	3.0 ± 0.1^b^	3.4 ± 0.1

BMC, bone mineral content; BMD, bone mineral density; sBMD, specific bone mineral density.

No. per genotype and sex: n = 9–11. Data are presented as mean ± standard error of mean. Student’s *t*-test vs. heterozygous mutants as controls: ^a^*P* < 0.05, ^b^*P* < 0.01, ^c^*P* < 0.001.

### Eye analysis

Slit lamp biomicroscopy, funduscopy and laser interference biometry (n = 15 per genotype and sex) were carried out at 16 weeks of age to analyze for anterior segment abnormalities, posterior segment abnormalities, and axial eye length abnormalities, respectively. Slit lamp biomicroscopy detected no abnormalities like nuclear flecks, corneal erosions or microphthalmia. Funduscopy also detected no abnormalities like microflecks or vessel alterations. Pigment patches and vessel attenuation were found in all mice of all four groups. Homozygous mutants vs. heterozygous mutants as controls also showed no difference in the axial eye length (3.6 ± 0.004 mm vs. 3.6 ± 0.004 mm in males, 3.6 ± 0.007 mm vs. 3.6 ± 0.004 mm in females) and in the ratio of axial eye length and body length (0.035 ± 0.0002 vs. 0.035 ± 0.0002 in males, 0.035 ± 0.0002 vs. 0.035 ± 0.0002 in females). Thus, no genotype-specific differences were found between homozygous mutants and heterozygous mutant controls. Line *Slc12a1*^*I299F*^ is based on the C3H genetic background and therefore showed the typical phenotype of retinal degeneration of C3H mice leading to blindness in the early phase of life in all mice irrespective of the genotype for *Slc12a1*. Therefore, no vision analysis was carried out.

### Energy metabolism

Homozygous mutants and heterozygous mutants as controls at the age of 17 weeks exhibited similar plasma cholesterol (154 ± 4 mg/dl vs. 165 ± 5 mg/dl in males, 117 ± 4 mg/dl vs. 118 ± 3 mg/dl in females) and triglycerides (349 ± 32 mg/dl vs. 427 ± 28 mg/dl in males, 288 ± 26 mg/dl vs. 256 ± 19 mg/dl in females) values.

Metabolic analysis was carried out with 23- to 24-week-old mice (n = 7 per genotype and sex) under *ad libitum* feeding conditions (Table 4). Homozygous mutant mice exhibited significantly less body weight than heterozygous mutants but assimilated comparable amounts of energy. In total, no clear differences in addition to the altered body weight parameters were observed for homozygous mutant mice compared to heterozygous mutant mice.

**Table 4:** **Analysis of energy metabolism of line** ***Slc12a1***^***I299F***^

	**Males**		**Females**		
**Parameter**	**Homozygous mutants**	**Heterozygous mutants**	**Homozygous mutants**	**Heterozygous mutants**	***t*-test**
Body weight (g)	33.0 ± 0.4	40.0 ± 1.1	31.7 ± 1.3	36.2 ± 1.3	c
Rectal body temperature (°C)	36.2 ± 0.04	36.3 ± 0.1	36.7 ± 0.1	36.6 ± 0.1	
Food intake (g/day)	3.7 ± 0.2	4.1 ± 0.1	3.9 ± 0.2	3.9 ± 0.1	
Energy uptake (kJ/day)	66.2 ± 4.2	72.6 ± 1.6	69.9 ± 4.0	70.1 ± 2.0	
Energy uptake / body weight (kJ/g/day)	2.0 ± 0.1	1.8 ± 0.1	2.2 ± 0.1	2.0 ± 0.1	a
Feces production (g/day)	0.91 ± 0.04	0.91 ± 0.05	0.81 ± 0.08	0.93 ± 0.02	
Energy content of feces (kJ/g)	15.3 ± 0.1	15.4 ± 0.1	15.3 ± 0.1	15.2 ± 0.1	
Metabolized energy (kJ/day)	51.3 ± 4.2	57.5 ± 1.7	56.4 ± 3.5	54.9 ± 1.7	
Metabolized energy / body weight (kJ/g/day)	1.55 ± 0.12	1.45 ± 0.06	1.78 ± 0.09	1.53 ± 0.07	
Food assimilation coefficient (%)	77.0 ± 1.7	79.1 ± 1.1	80.7 ± 1.8	78.3 ± 0.4	

23-24 week-old mice (n = 7 per genotype and sex) were tested under *ad libitum* conditions. Data are presented as mean ± standard error of mean. Student’s *t*-test: homozygous mutants (males and females) vs. heterozygous mutants (males and females) as controls: a, *P* < 0.05; c, *P* < 0.001.

### Immunology

Peripheral blood leukocytes were isolated and viable cells were subsequently analyzed for the identification of main cell populations (B cells, T cells, granulocytes, NK cells) und subpopulations. Plasma antibody levels were determined simultaneously in the same samples (Table 5). The initial analysis of homozygous mutants vs. heterozygous mutant controls at the age of 17 weeks revealed some changes affecting male homozygous mutants. However, they were not found in blood samples from the same cohort of mice at the age of 22 weeks (not shown). Also no changes in autoantibodies (anti-DNA antibodies, rheumatoid factor) were observed (not shown).

**Table 5:** **Immunology analysis of line** ***Slc12a1***^***I299F***^

	**Males**		**Females**	
**Parameter**	**Homozygous mutants**	**Heterozygous mutants**	**Homozygous mutants**	**Heterozygous mutants**
CD3^+^	38.8 ± 1.8	35.1 ± 2.3	37.4 ± 1.7	36.1 ± 1.3
CD4^+^	25.7 ± 1.2	22.9 ± 1.6	23.3 ± 1.3	21.9 ± 1.1
CD8β^+^	22.2 ± 0.9	20.6 ± 1.6	22.9 ± 1.0	22.4 ± 1.0
CD19^+^	28.9 ± 1.1	31.1 ± 1.1	30.3 ± 1.1	29.6 ± 1.0
CD19^+^CD5^+^	3.5 ± 0.2	3.3 ± 0.2	3.9 ± 0.2	3.5 ± 0.1
CD19^+^CD5^−^	96.4 ± 0.2	96.5 ± 0.2	95.9 ± 0.2	96.3 ± 0.1
CD49b^+^	29.9 ± 1.5^b^	23.0 ± 1.6	28.8 ± 1.9	27.3 ± 1.6
Gr1^+^	31.7 ± 0.8	37.0 ± 2.1	27.0 ± 1.4	30.0 ± 1.3
IgA	445 ± 14	453 ± 40	475 ± 18	478 ± 17
IgG1	130 ± 12	158 ± 17	174 ± 13	211 ± 20
IgG2a	385 ± 25	476 ± 34	558 ± 43	518 ± 35
IgG2b	94 ± 4^a^	147 ± 21	175 ± 10	165 ± 8
IgG3	324 ± 25^a^	424 ± 18	465 ± 25	408 ± 22
IgM	1338 ± 142	1782 ± 275	1623 ± 164	1590 ± 195

Data are frequencies of main leukocyte subsets in blood (%) and concentration (μg/ml) of antibodies of different isotypes in plasma.

17-week-old mice were tested. No. per genotype and sex: n = 15. Data are presented as mean ± standard error of mean. Student’s *t*-test vs. heterozygous mutants as controls: ^a^*P* < 0.05, ^b^*P* < 0.01.

The detailed examination of cellular subsets bearing activation/memory markers in blood samples of homozygous mutants and heterozygous mutant controls at the age of 17 weeks and 22 weeks showed an increase of activated/memory CD4^+^CD62L^low^ helper T cells (*P* < 0.05 at the age of 17 weeks and 22 weeks) and CD8^+^ CD62L^low^ cytotoxic T cells (*P* < 0.05 at the age of 22 weeks) in homozygous mutant mice. Thus, this mild but steadily expressed phenotype was confirmed (not shown) which fits to clinical data from human patients with chronic renal failure, where changes in memory T cells bearing the CD62L marker are well described [29]. It has been established for humans that changes affecting frequencies of leukocyte subsets are secondary effects of renal diseases.

### Lung function

Spontaneous breathing patterns during sleep, rest and activity were analyzed in 5–6 mice per genotype and sex at 21 weeks of age by whole body plethysmography (Table 6). Homozygous mutant males exhibited significantly less body weight than heterozygous mutant males as controls, whereas homozygous mutant females showed analogous results by tendency. The mean of all breathing frequencies (mean f) measured during the 40-minute examination period was calculated as a general parameter to assess whether the duration of rest and activity was similar in all groups. It did not differ between homozygous mutants and heterozygous mutants. Specific tidal volumes and specific minute ventilations (sTV and sMV) during sleep, rest and activity, respectively, were calculated by relating the absolute values to the body weight of the animals. Overall, observed differences between homozygous mutants and heterozygous mutant controls were within the physiological ranges and of minor relevance. This was confirmed by comparison of the data to a cohort of C3H mice taken from a different breeding colony and maintained under different housing conditions (not shown). Therefore it was concluded that the mutation *Slc12a1*^*I299F*^ does not affect the respiratory system.

**Table 6:** **Analysis of lung function of line** ***Slc12a1***^***I299F***^

	**Males**		**Females**	
**Parameter**	**Homozygous mutants**	**Heterozygous mutants**	**Homozygous mutants**	**Heterozygous mutants**
Body weight (g)	33.6 ± 0.8 ^a^	38.9 ± 1.5	34.8 ± 1.4	36.3 ± 2.0
Mean f (1/min)	334 ± 19	354 ± 7	355 ± 23	308 ± 25
Sleep f (1/min)	125 ± 5 ^b^	149 ± 4	130 ± 7	128 ± 6
Rest f (1/min)	277 ± 19	297 ± 4	293 ± 11^a^	251 ± 13
Activity f (1/min)	468 ± 6	474 ± 11	460 ± 5	426 ± 20
Sleep sTV (μl/g)	8.4 ± 0.2	7.3 ± 0.4	9.4 ± 0.5	8.5 ± 0.6
Rest sTV (μl/g)	6.0 ± 0.2	5.5 ± 0.3	6.5 ± 0.3	6.3 ± 0.3
Activity sTV (μl/g)	5.8 ± 0.2	5.5 ± 0.4	6.2 ± 0.2^a^	5.5 ± 0.2
Sleep sMV (ml/min/g)	1.0 ± 0.04	1.1 ± 0.1	1.2 ± 0.04	1.1 ± 0.1
Rest sMV (ml/min/g)	1.5 ± 0.1	1.5 ± 0.1	1.8 ± 0.1^b^	1.4 ± 0.04
Activity sMV (ml/min/g)	2.7 ± 0.1	2.5 ± 0.2	2.8 ± 0.1^b^	2.3 ± 0.1

f, respiratory rates; mean f (1/min), the mean of all breathing frequencies (mean f) measured during the 40-minute examination period was calculated as a parameter to assess whether the duration of rest and activity was similar in all groups; sTV, specific tidal volumes and sMV, specific minute ventilations were calculated by relating the absolute values to the body weight of the animals.

21-week-old mice were tested. No. per genotype and sex: n = 5–6. Data are presented as mean ± standard error of mean. Student’s *t*-test vs. heterozygous mutants as controls: ^a^*P* < 0.05, ^b^*P* < 0.01.

### Neurology

Basic neurological functions were tested in 16-week-old mice (n = 15 per sex and genotype) by modified SHIRPA and forelimb grip strength analysis for the assessment of muscular function.

The modified SHIRPA protocol is a semi-quantitative screening method for the overall qualitative analysis of abnormal phenotypes in mice and includes 23 test parameters each contributing to the overall assessment of general health, posture and movement, autonomic reflexes, as well as behavioural aspects. The body weight was significantly decreased in homozygous mutants vs. heterozygous mutants as controls (31.2 ± 0.4 g vs. 35.6 ± 0.7 g in males, and 30.7 ± 0.9 g vs. 33.2 ± 0.9 g in females; *P* < 0.05). All other SHIRPA parameters (body position, tremor, palpebral closure, coat appearance, whiskers, lacrimation, defecation, transfer arousal, locomotor activity, gait, tail elevation, touch escape, positional passivity, skin colour, trunk curl, limb grasping, pinna reflex, corneal reflex, righting reflex, contact righting, evidence of biting, vocalisation) were without significant alterations in homozygous mutants vs. heterozygous mutant controls. Also forelimb grip strength did not differ between both genotypes.

### Nociception

Analysis of nociception was carried in 18-week-old mice by using the hot plate assay at 50°C for the detection of altered pain response. The reactions due to thermal pain by shaking and licking of the hind paws were measured in the homozygous mutants and heterozygous mutant controls (latency in seconds for shacking of hind paws: 46.5 ± 2.7 vs. 46.5 ± 2.7 in males, 35.3 ± 2.6 vs. 43.1 ± 2.8 in females (*P* < 0.05); latency in seconds for licking of hind paws: 51.6 ± 2.5 vs. 50.3 ± 2.5 in males, 39.8 ± 2.4 vs. 48.6 ± 2.7 in females (*P* < 0.05); n = 10–15 per genotype and sex). Thus, slight differences were found in homozygous mutant females showing mild hyperalgesia by a decreased latency period.

## Discussion

*Slc12a1*^*I299F*^ mutant mice were examined using a systemic and standardized, comprehensive phenotypic analysis at the age of 14–26 weeks. In humans, nephropathy-causing reduced ion transport capacity of the NKCC2 dimers occurs if both alleles are mutated [2], and the recessive character of *Slc12a1* mutations to cause renal disease has been shown in knockout mice [23] as well as in our previous publication of line *Slc12a1*^*I299F*^[22]. Thus, *Slc12a1*^*I299F*^ homozygous mutants were examined and *Slc12a1*^*I299F*^ heterozygous mutant littermates were used as control animals. NKCC2 is a 12-transmembrane cotransporter. The point mutation of line *Slc12a1*^*I299F*^ leads to the amino acid exchange I299F that is located at the first position of the fourth transmembrane-spanning domain. This region is well conserved among mammalian species [22]. In humans, disease-causing mutations are described in this region, e.g. affecting the amino acid directly before I299 (http://www.hgmd.org). The most obvious phenotype of *Slc12a1*^*I299F*^ homozygous mutants was a severe defect in urine concentrating ability, leading to increased urine excretion and water consumption. In addition, homozygous mutants exhibited hypotension and osteopenia due to their impaired electrolyte homeostasis [22].

The systemic and standardized phenotypic analysis of line *Slc12a1*^*I299F*^ in the German Mouse Clinic is described to evaluate further primary effects of this mutation as NKCC2 has been shown in rodents to be expressed in other tissues i.e. the gastrointestinal tract [5], pancreatic beta cells [6], and specific compartments of the ear [7], nasal tissue [8] and eye [9]. In addition to the cardiovascular and skeletal alterations, the impaired kidney function may also lead as secondary consequences to alterations in hematological parameters, chronic inflammation of the gastrointestinal system, or uremic effects on the central nervous system. Furthermore, several studies on the effect of sex steroids on NKCC2 in rodent kidneys yielded variable results ([4] and refs therein), therefore sex-specific alterations may occur in the line *Slc12a1*^*I299F*^.

The analyses of the clinical chemistry, cardiovascular function and dysmorphology at additional time points supported the already published data of line *Slc12a1*^*I299F*^[22]. The hematological analyses at the age of 17 and 22 weeks revealed a mild erythropenic anemia in male but not female homozygous mutants. One possible explanation is that this might occur as a secondary effect due to the impaired kidney function which might also lead to a reduced erythropoietin production. Although the homozygous mutant mice exhibited severe polyuria, there was no hint for a general dehydration of the mice as it has been described in *Slc12a1* homozygous knockout mice at the perinatal stage [18]. The heterozygous mutant animals showed no obvious differences compared to a cohort of C3H wild-type mice from the same facility. Compared to the hypotension already described in 3-month-old *Slc12a1*^*I299F*^ homozygous mutants, 19-week-old homozygous mutants showed the same tendency of the data but without reaching statistical significance. Lung function data of homozygous mutants showed no obvious alterations.

In the morphological analysis, genotype-specific alterations were found. The maxillary incisors of most female homozygous mutants showed a pale colour, and the urine colour of female homozygous mutants was pale. To date, there is no published information regarding the expression of *Slc12a1* in teeth. At the age of 21 weeks, body weight and fat content were significantly lower in homozygous mutants. The lower level of body weight was confirmed in further analyses in the German Mouse Clinic at 21 and 23–24 weeks of age. Homozygous mutants only exhibited 83-86% (males) and 88-96% (females) of the body weight of the heterozygous mutant controls. A lower body weight has been already found by tendency in 3-month-old *Slc12a1*^*I299F*^ homozygous mutants [22]. Analysis of the energy metabolism at the age of 23–24 weeks under *ad libitum* conditions revealed a significantly lower body weight of homozygous mutant mice, but no clear additional differences. Thus, the metabolic and thermoregulatory properties have to be clarified in further studies. Analyses of the sensory organs showed no obvious effect of the mutation on hearing ability, olfaction or on eye development in homozygous mutants vs. heterozygous mutant controls.

The immunology analysis revealed mild deviations in the homozygous mutants, most likely as a secondary effect of the primary renal impairment. No indications were observed for the appearance of major inflammatory processes in the mutant mice. In the allergy analysis, also no genotype-specific differences were found (data not shown). The neurological results of homozygous mutants were within the physiological range of C3H mice and did not permit to define an altered behavioral pattern for the homozygous mutant animals. Mental retardation has been described in severely affected human patients [10]. The analysis of nociception by thermal pain at the age of 18 weeks found slight differences in homozygous mutant females showing mild hyperalgesia.

## Conclusions

*Slc12a1* is mainly expressed in the kidney, and amino acid-changing mutations of *SLC12A1* in humans lead to the severe defect in the ability to concentrate urine. The systemic phenotypic analysis of *Slc12a1*^*I299F*^ homozygous mutant mice vs. heterozygous mutant mice as controls on the C3H inbred genetic background at the age of 14–26 weeks reproducibly revealed the polyuric nephropathy, impaired electrolyte homeostasis, hypotension and osteopenia. *Slc12a1*^*I299F*^ homozygous mutant mice showed a significantly lower body weight and fat content. This might reflect a decreased activity of the mutant SLC12A1 in the gastrointestinal tract and/or pancreatic beta cells and/or might be a secondary effect of the kidney disease. No obvious effect of the mutation was found on hearing ability, olfaction or on eye development. The other analyses showed small additional effects. Thus, depending on the genetic background, long-term effects additional and/or secondary to the kidney lesions might also appear in humans harboring *SLC12A1* mutations.

## Competing interests

All authors have no competing financial or other interests in relation to the manuscript.

## Authors’ contributions

Conceived and designed the experiments: EK BR LB IB DHB CD RE JF JG WH BI SK TK IR JR AS HS AZ HF VGD MHdA EW BA. Performed the experiments: EK BR LB IB CD RE JF JG WH SK IR JR AS HF. Analyzed the data: EK BR LB IB CD RE JF JG WH SK IR JR AS HF BA. Contributed reagents/materials/analysis tools: EK BR LB IB DHB CD RE JF JG WH BI SK TK IR JR AS HS AZ HF VGD MHdA EW BA. Wrote the manuscript: EK BA. All authors read and approved the final manuscript.

## Additional file

## Supplementary Material

Additional file 1: **Time points of the phenotypic analyses described for the line** ***Slc12a1***^***I299F***^**in the German Mouse Clinic (GMC).** Click here for file
