# Supplementary material for: Standardized, systemic phenotypic analysis of Slc12a1I299F mutant mice
Source: J Biomed Sci. 2014 Aug 2;21(1):68. doi: 10.1186/s12929-014-0068-0 (PMC4237776; doi:10.1186/s12929-014-0068-0)
Supplement: Additional file 1: — Time points of the phenotypic analyses described for the line Slc12a1 I299F in the German Mouse Clinic (GMC). [file s12929-014-0068-0-S1.docx]

Supplementary Table S1. Time points of the phenotypic analyses described for the line *Slc12a1^I299F^* in the German Mouse Clinic (GMC)

| Phenotypic analysis |  | Line *Slc12a1^I299F^* |
| --- | --- | --- |
| Start and end of the GMC analysis |  | 14-26 weeks p.p. |
| Clinical chemistry | plasma analysis | 17 and 22 weeks |
|  |  | previously published data: 7 weeks, 3, 6 and 12 months ^1^ |
|  | urine analysis | previously published data: 3 months ^1^ |
| Hematology |  | 17 and 22 weeks |
|  |  | previously published data: 3 months ^1^ |
| Cardiovascular analysis | heart rate, blood pressure | 19 weeks |
|  | electrocardiography | 20 weeks |
|  | heart rate, blood pressure, Nt-proANP | previously published data: 3 months ^1^ |
| Dysmorphology | clickbox test | 14 weeks |
|  | visual inspection | 14 weeks |
|  | X-ray | 21 weeks |
|  | bone metabolism | previously published data: 9 months ^1^ |
| Eye analysis |  | 16 weeks |
| Energy metabolism | plasma lipids | 17 weeks |
|  | metabolic analysis | 23-24 weeks |
| Immunology |  | 17 and 22 weeks |
| Lung function |  | 21 weeks |
| Neurology |  | 16 weeks |
| Nociception |  | 18 weeks |
| Pathology | kidney | previously published data: 3-18 months ^1^ |
| Allergy |  | 17 weeks (no obvious differences; not shown) |
| Transcriptome analysis of the kidneys |  | 26 weeks (no clear differences; not shown) |

The GMC analyses were carried out in homozygous mutants and heterozygous mutants as controls.

Nt-proANP, plasma concentration of the N-terminal fragment of the pro-atrial natriuretic peptide.

^1^ Cited data that was previously published (Kemter E, et al. (2010) Am J Physiol Renal Physiol 298: F1405-F1415). These results were derived from analyses of homozygous mutants, heterozygous mutants, and wild-type littermates as controls.
